# Mutational Analysis of *Angiogenin* Gene in Parkinson's Disease

**DOI:** 10.1371/journal.pone.0112661

**Published:** 2014-11-11

**Authors:** Meng-Ling Chen, Ruey-Meei Wu, Chun-Hwei Tai, Chin-Hsien Lin

**Affiliations:** 1 Department of Neurology, National Taiwan University Hospital, College of Medicine, National Taiwan University, Taipei, Taiwan; 2 Department of Life Science, National Taiwan University, Taipei, Taiwan; Nanjing Medical University, China

## Abstract

Mutations in the angiogenic factor, angiogenin (*ANG*), have been identified in patients with both familial and sporadic amyotrophic lateral sclerosis (ALS) and are thought to have a neuroprotective function. Parkinsonism has been noted in kindreds with *ANG* mutations and variants in the *ANG* gene have been found to associate with PD in two Caucasian populations. We therefore hypothesized that mutations in *ANG* may also contribute to idiopathic Parkinson's disease (PD). We sequenced *ANG* gene in a total of 1498 participants comprising 750 PD patients and 748 age/gender matched controls from Taiwan. We identified one novel synonymous substitution, c.C100T (p.L10L), in a single heterozygous state in one PD patient, which was not observed in controls. The clinical phenotypes and [^99m^Tc]-TORDAT-SPECT images of the p.L10L carrier were similar to that seen in idiopathic PD. In addition, we also identified one common variant, c.T330G (p.G110G, rs11701), which was previously reported to associate with PD risk in Caucasians. However, the frequency of TG/GG genotype was comparable between PD cases and controls (odds ratio: 0.85, 95% confidence interval: 0.29–2.55, P = 0.78). Our results did not support that *ANG* rs11701 variant is a genetic risk factor for PD in our population. We conclude that mutations in *ANG* are not a common cause for idiopathic PD.

## Introduction

The potent angiogenic factor, angiogenin (*ANG*), has been known to associate with both familial and sporadic Amyotrophic Lateral Sclerosis (ALS) [1], [2]. Angiogenin protein, which involved in neovascularization, also have neurotrophic and neuroprotective functions, further supporting a role of this protein in the neurodegenerative disorder [3]. Notably, studies have shown that several ALS patients carrying *ANG* mutations also demonstrated signs of Parkinsonism [4]. Epidemiological studies showed that ALS patients are at increased risk of developing Parkinson's disease (PD) [5], suggesting there is a genetic link between ALS and PD.

The significant association between *ANG* genetic variants and risk of PD has recently been confirmed in two case-control studies in Caucasian populations, in which the frequency of *ANG* variants in PD patients was 0.46%–0.63% in the European-American PD cases but less than 0.05% in control subjects [6], [7]. Experimental studies have shown levels of angiogenin are reduced in a transgenic α-synuclein PD mouse model and exogenous angiogenin protects neuronal loss in 1-methyl-4-phenylpyridine (MPP+) treated cellular models of PD [8], [9]. These findings reinforce the possibility that mutations or variants of *ANG* may contribute to the disease susceptibility of PD.

We have previously performed comprehensive mutation analysis of multiple candidate genes in a cohort of PD patients from Taiwan [10]–[13]. However, the major genetic causes in most PD patients, especially early-onset ones, are still unclear.

Given the recent evidence that *ANG* genetic variants are involved in PD, we performed mutational screening of *ANG* in a large cohort of PD cases and controls subjects.

## Methods

A total of 1498 study participants including 750 PD patients and 748 age/gender matched control subjects were included in this study. Among PD patients, 50 had a family history of PD, 500 were sporadic late-onset PD and 200 were early-onset PD patients (onset age less than 50 years). Mutations in the *a-synuclein*, *Parkin*, *PINK1*, *DJ-1*, *LRRK2*, *ATP13A2*, *HTRA2*, *SCA2*, *SCA3* and *C9Orf72* genes were previously excluded in all familial and early-onset PD patients [10]–[15]. The diagnosis of PD was based on the UK PD Brain Bank clinical diagnostic criteria [16]. Unrelated adult volunteers without neurological disease were recruited as controls from the community and from our hospital. The study was approved by the institutional ethics board committee of National Taiwan University Hospital and written informed consent forms were taken from all the study participants.

DNA extraction from venous blood was performed using standard protocols. The entire gene of *ANG* was sequenced as methods described before [7].

Hardy-Weinberg equilibrium (HWE) and chi-square test for genotype frequency in cases and controls were examined. Logistic regression was used to test for association between genotypes and PD under an additive model, with the homozygotes for the more common allele were used as the baseline risk group. Power calculations were done using Genetic Power Calculator [17]. The prevalence of PD in Taiwan is estimated to be 130 cases per 100,000 individuals [18], and the odds ratios (ORs) for each risk allele of the tested genetic variants was approximately 1.4 as estimated in previous studies [6]. Statistical analysis was performed using the STATA, version 8.0 (Texas, USA).

## Results

Demographic data for PD patients are mean age at symptom onset was 57.1±11.9 years (range 18–85 years), and the age at the enrollment was 67.7±11.6 years (range 33–95 years). There were 375 men and 375 women. The genetic power of our study is 89%.

We identified one synonymous substitution, p.L10L, in a single heterozygous state in one PD patient but not in 748 controls. The clinical phenotypes and [^99m^Tc] TRODAT-SPECT images were similar to those in idiopathic PD. We also found one common variant, c.T330G (p.G110G, rs11701), in our patients and controls. This variant was previously observed to associate with risk of ALS first [1], and then risk of sporadic PD in a Caucasian population [6]. However, we noted that the frequency of TG/GG genotype was comparable between PD cases and controls (0.9% vs. 1.0%, Table 1). The clinical phenotypes were similar between carriers and non-carriers.

**Table 1:** Distribution of *ANG* rs11701 variant and estimated odds ratio in relation to PD risk.

	PD patients N = 750	Control N = 748	OR (95% CI)	*P* value
rs11701:c.T330G(p.G110G)				
*TT*	743 (99.1)	740 (99.0)	1.00	
*TG*	6 (0.8)	7 (0.9)	0.85 (0.29–2.55)	0.78
*GG*	1 (0.1)	1 (0.1)	0.99 (0.06–15.95)	0.99

PD: Parkinson's disease; OR: odds ratio; CI: confidence interval.

## Discussion

We present a comprehensive mutational analysis of the *ANG* gene in a large cohort of PD patients and control subjects. We did not identify any pathogenic mutations. Heterozygous genetic substitutions were present in approximately 1.0% of PD cases, but no clearly risk variants were identified.

*ANG* encodes a 123-residue protein, which is synthesized with a signal peptide of 24 amino acids, that is cleaved to form the mature protein. The angiogenin protein is thought to be involved in a number of biological processes, including neovascularization, RNA metabolism, neurite outgrowth, axonal guidance, and is also a neuroprotective factor [19]. Recently, angiogenin has been linked to PD. The *in vitro* studies showed that angiogenin reduces neuronal death in MPP^+^ treated human dopaminergic cell line models through activating the Akt survival signaling pathway [9]. In addition, two genetic screens showed several *ANG* variants to be associated with PD [6], [7]. One study conducted in an American cohort enrolling 630 PD patients and 676 controls and found the frequency of *ANG* variants were 0.63% in PD patients and zero in controls [6]. Another large-scale study of mixed American and European samples enrolling 3146 PD patients and 7668 control subjects found that the frequency of *ANG* variants was 0.45% in PD patients and 0.04% in control subjects [7]. These previously reported exonic genetic variants of *ANG* were summarized in Table 2. These observations lead to the speculation that *ANG* genetic variants may increase the risk of PD, especially the rs11701 variant. However, our results did not support the above-mentioned studies that we did not find an increased risk for PD in rs11701 variant carriers compared to non-carriers. Compared to the frequency of *ANG* variants were 0.45%–0.63% in Caucasian PD cases [6], [7], the frequency of *ANG* variant was 0.9–1.0% in our population, although one previous study in another Chinese population reported the frequency of *ANG* variants were zero [20]. The relatively low frequency of *ANG* variants in our ethnicity suggests an ethnic difference effect of this candidate gene. To the best of our knowledge, our study is the first large-scale survey of ANG gene in Asians and could provide a sufficient power to show that ANG is unlikely to play a major role in PD risk in our ethnicity. Further studies in other ethnic cohorts will be important to address the potential pathophysiologic role of ANG in PD.

**Table 2:** Exonic variants of *ANG* that has been identified in PD patients in previous literature.

Variants	Identified ethnicity	MAF	OR (95% CI)	*P* value	References
p.H13R	Germany	0.001	NA	NA	Van Es et al., 2011 [7]
p.K17I	Germany, Netherlands, US	0.003	NA	NA	Van Es et al., 2011 [7] and Rayaprolu et al., 2012 [6]
p.D22V	Netherlands	0.001	NA	NA	Van Es et al., 2011 [7]
p.K54R	Netherlands	0.001	NA	NA	Van Es et al., 2011 [7]
p.K60E	US	0.001	NA	NA	Rayaprolu et al., 2012 [6]
p.Q77P	US	0.001	NA	NA	Rayaprolu et al., 2012 [6]
p.R95Q	Netherlands	0.001	NA	NA	Van Es et al., 2011 [7]
p.G110G	US	0.125	1.40 (1.08–1.80)	0.01	Rayaprolu et al., 2012 [6]
	Taiwan	0.01	0.85 (0.29–2.55)	0.78	The current study
P.R121C	Italy	0.001	NA	NA	Van Es et al., 2011 [7]

PD: Parkinson's disease; MAF: minor allele frequency in PD cases; OR: odds ratio; CI: confidence interval; NA: not available.
